# Sketch of Dupuytren

**Published:** 1846-09

**Authors:** 


*Medical Gallery.*—The Medico-Chirurgical Review, for July, contains a notice of a work by Reveillé-Parise, one of the chapters of which presents, among many others, a sketch of the life and character of the great French surgeon Dupuytren, which we are sure will be read with pleasure.

The determined and overbearing character of this celebrated surgeon manifested itself from the very commencement of his career. When, at seventeen years of age, he was appointed one of the Demonstrators at l'Ecole de Medicine, his poverty was so great that he was obliged to mend his own clothes. So far from despairing, however, he had such a complete conviction of his own superiority, and presentiment of its successful exertion, that he had already in view the surgical dictatorship which he afterwards attained. The renown of Bichat was a source of continual vexation to him, and upon hearing of his death, he exclaimed, "I begin to breathe." From this period, too, his reputation began to increase rapidly.

Knowledge, judgment, indefatigable industry, untiring patience, address, energy, and resolution, were all employed by him in the manner characteristic of a man who is determined to succeed—one who is never at ease but at the head of the crowd, who proceeds straight to the end in view, in spite of all the obstacles and difficulties of rivalry. This is in fact the period at which Dupuytren really worked, when he amassed most of his scientific labors, although it was long before he reached his eventual eminence. There was then to be observed in him the decided desire of increasing the riches of science, of extending its limits, united with that burning ambition which destroys rest, and which is the foundation of that strength which characterizes the man impatient of all control and jealous of every influence.

His avowed determination in 1812, to obtain the professorship beld by Sabatier in l'Ecole de Medicine raised him numerous enemies and opponents; but he triumphed over all, in spite of an evident desire of the judges to favor some of these. Nothing now impeded his progress.

Arrived at the apogee of his renown, the genius of Dupuytren was quite equal to his position; and he was enabled to struggle with the torrent of occupations by which he was surrounded. There was indeed something really gigantic in that intelligence which provided for everything by its prodigious activity, indefatigable industry, determined arduor, and by a sort of ubiquity which allowed him to accomplish as well as to desire everything. For twenty years he was always one of the earliest at the Hôtel Dieu, however inclement the season. He passed more than five hours there in the severest bodily and mental exertion. No sooner had he left these, than new duties and crowds of patients called him to all parts. * * * * * Whoever wishes to give unrestricted praise to Dupuytren, must consider him in his professor's chair. There he triumphed—there he excelled. It was a remarkable thing to hear this illustrious surgeon delivering his beautiful lectures on Clinical Surgery, amidst three or four hundred silent and attentive pupils. With what order and method he enchained the attention of his auditors! how accomplished was he in instructing them, exhibiting the light of evidence with a skilful and vigorous logic, and inculcating good and sound information! His clear, calm, strong delivery, lucid and expressive, fluent, easy without prolixity, elegant without effort, always fortified by things and facts; the clearness of his views; his rare talent for exposition and deduction; his astonishing facility in defining, classifying, and expressing his ideas, in rendering objects sensible and evident, and in arresting, fixing, and directing the attention, gave to his lectures a singular attraction. No idle detail, declamation, or ornament was there: all was clear, precise, intelligible; all tended to fact, to its object, and to instruction. * * * * * * Nevertheless, the pupils sometimes reproached the professor with dwelling only with predilection upon those cases in which success had crowned his efforts, and disguising his reverses with skill. Another, and perhaps better-founded, reproach is, that this illustrious professor never quoted any one else, and French surgeons still less than any. To speak the truth, he had a marked and exclusive disdain for all that had not proceeded from himself; and this indifference, or rather scepticism for everything and everybody, when the talent of his rivals was in question, had its root in his naturally haughty character—completely despising the opinions of others—those of the past as useless, and those of the present as inferior.

What I have said of the professor exhibits his qualities as a surgeon. It is allowed by all that Dupuytren was the most remarkable man in the profession of his epoch. Few have possessed that vast and rare assemblage of qualities which constitute a great surgeon. He had especially an admirable strength of judgment, which rendered his diagnosis so true and affirmatory, that it struck every one with astonishment. Moreover, in the observation and application of precepts, there was an exactness of analysis, a certainty of practical good sense, and a depth of examination, which are certainly rare. Although bold, without the least trace of pusillanimity, he never operated until the last moment, when he was certain that this furnished the last resource of art. * * * * * * Arrived at the height of his reputation, Dupuytren desired to be not the chief but the tyrant of French surgery; and the most exclusive and bitter individuality dominated his entire being, dictated his thoughts and governed his actions. So, in his social relations, he much preferred unexacting friendships and passive unconditional devotion.

Justice obliges us to add, that the high faculties of this great surgeon were not those which may be termed transmissible. He had not a genius for discovery, but for application. This leaves but little harvest for succeeding generations to reap. From such great talent and renown one would expect an imposing succession of works which would add to the acquirements of science, and long leave their traces there. Alas! what remains to us of Dupuytren, what his biographers will carefully collect, is not to be compared with that which we had a right to hope for. It is this which has given rise to the saying that, after all, this great man was only a famous man. * * * * * * In studying this celebrated surgeon with care, we may, to a certain extent, explain this defect of accordance between his labors and talent, and the insignificant amount of results. He possessed much more of the talent for teaching, and all that required the aid of speech and action, than for slow, continuous labor. Next, he had embraced the whole circle of surgery. He was not one of those who devote their lives to the production and realization of an idea. He wished to know and distinguish himself in every part of the profession, whence it resulted that he only contributed here and there to the progress of some portions of surgery. Add to this, that desirous of becoming the first in all, he wished to accomplish this by the aid of a large fortune, and amassing this occupied him incessantly, especially as this was a natural bent of his disposition.

A lofty pride seemed to have been his distinguishing feature, which accompanied him in every act and word, and the inordinate ambition hence arising doubtless was one of the causes of his merit and renown. To serve his ambition, however, he would condescend to almost any meanness and duplicity, so as to impart to his character, observed for any length of time, the most strange contrasts. When his object was to be answered by it, it was painful to see "the insinuating air, the wheedling suppleness of so proud and irritable a man." He was surrounded by flatterers, but he was also exceedingly susceptible to the attacks and criticisms his own disdainful conduct provoked.

Criticism irritated him as an injury, while blame tormented him as an intolerable novelty. Admirers and acquaintances he had, but few or no friends, and found the surgical crown, like others, was not without its thorns. His genius, talent, and dignities were envied, but no one was ignorant that beneath this brilliant surface were anguish and chagrin. He was looked upon as a sort of power whom one must prevent from becoming an enemy, but with the impossibility of ever becoming his friend. Every one stretched a hand to him, but no one shook his cordially. He was at once admired, feared, and blamed—a singular destiny for a man of such incontestible superiority. Let us add that, like other proud, susceptible, and irritable beings, he was unequal and changeable. Literally, to use the vulgar expression, one did not know which end to lay hold of him by. He did good and evil by starts and caprice. If, in some cases, he might be taxed with falsehood and harshness, in others he exhibited an exemplary loyalty and unheard of disinterestedness.

With all his affectation of simplicity he coveted scientific distinctions, and was especially fond of his title of *Baron.* Probably from the obstacles to his progress which he found his poverty to offer in early life, he acquired an insatiable love for money, and became immensely rich. Having heard that Astley Cooper possessessed six millions of francs he would never rest until he had accumulated the like sum. To this end he sacrificed all the the time that might have been devoted to the advancement of science; and even his Clinical Lectures would not have been preserved but for the care of some of the auditors. Dupuytren himself published nothing, except a wretched letter in the newspapers of 1832, announcing decoction of poppy-heads a cure for the cholera! His riches, however, brought no satisfaction, but only the desire to add to them; he was not organized for happiness and never possessed it. He died aged 58. His will, M. Parise says, does high honor to him, but he does not state its dispositions.

